# Supplementary material for: JBrowse 2: a modular genome browser with views of synteny and structural variation
Source: Genome Biol. 2023 Apr 17;24:74. doi: 10.1186/s13059-023-02914-z (PMC10108523; doi:10.1186/s13059-023-02914-z)
Supplement: Supplementary file 2 — Additional file 2. Review history. [file 13059_2023_2914_MOESM2_ESM.docx]

Review History

**First round of review**

**Reviewer 1**

**Were you able to assess all statistics in the manuscript, including the appropriateness of statistical tests used?** There are no statistics in the manuscript.

**Were you able to directly test the methods?** Yes.

**Comments to author:**

The jBrowse software has been instrumental for thousands of genome analysis projects. it is *the* standard software for visualising new genome data, given its flexibility and applicability for a large range of species. It has been previously published. Here the authors present a major update to this software. These updates include:

- the ability to install via a point-and-click interface

- multiple visualisations that make inter genome-comparisons. The figures in the paper illustrate this nicely.

- performance and flexibility upgrades (e.g. for facilitating further customisation).

The upgrades are well worthwhile. I think this does represent the type of major changes that do warrant new publication. I expect that this will further increase the relevance of the tool. I thus expect that it will continue to be the reference for further genomic projects.

The software is open source under the apache license.

The paper reads very well, and includes extensive benchmarking details which quantify the performance improvements. The software has been available online for a while. I have used it productively.

I feel that this paper can be accepted as is, and that the software will be of use to genome biologists across their broad range of interests

**Reviewer 2**

**Were you able to assess all statistics in the manuscript, including the appropriateness of statistical tests used?** There are no statistics in the manuscript.

**Were you able to directly test the methods?** Yes.

**Comments to author:**

The authors present JBrowse 2, a general-purpose genome browser. JBrowse 2 is a new version of the JBrowse genome browser widely used for visualizing data on a reference genome sequence.

The main novelty of JBrowse 2 is its updated codebase and design. It also features implementations of new views for structural variation analysis and comparative genomics, that complements the more classical linear genome browser views. In the manuscript, the authors:

Provide examples and use cases of these new views on example datasets. These datasets are made available through a web instance of the browser.

Outline several features to facilitate integration and sharing of data locally and remotely.

Provide a benchmark of rendering capability, in comparison to other JavaScript based genome-browsers.

Overall, my opinion of this work is positive. The manuscript is well written, the novel features are relevant and this new version of JBrowse will certainly prove at least as useful to the wider community as its predecessor. The comparative genomics features are similar to other alternatives, but they benefit from being integrated within a framework of an existing genome browser that is compatible with many datatypes.The JBrowse browser is available, among others, through a web interface and a desktop application. I was able to use both applications on the author-provided datasets, as well as custom local datasets, and did not encounter major issues. The desktop application was remarkably easy to install on a personal laptop and ran smoothly. The documentation available on the main website is complete.

I have few minor comments on the manuscript itself:

-The authors benchmarked the performance of JBrowse2 for rendering data using sequencing reads mapped to a reference genome sequence. They note their performances are comparable to IGV.js, another java-script based genome browser. It is arguably true from the data they provide, however, JBrowse2 appears to be more performant at displaying long read data but slightly less performant in rendering short read data than JBrowse 1 and igv.js. This detail does not affect the authors' conclusion but please elaborate on this difference.

-Two references cited from Bioarxiv (JBrose Jupyter and IGV.js) now have a peer-reviewed paper. Please update it.

As mentioned before, I tested the software on a few use cases, from the author-provided demo and from local data. I mainly focused on the comparative genomics features. I have a few comments and feedback to the authors. Given the scope of the project, I would not expect the minor comments to be addressed for this manuscript to be published. However, I believe it may help them improve these features in the future.

Note: All comments below result from tests on a personal computer (OS:Ubuntu 22.04; Opera Browser 95)

Dotplot synteny viewer:

-In both the desktop and browser version, this plot sometimes displays long margins at the end of the x axis and y axis when scrolling or zooming in and out, which can result in most of the view being occupied by empty space.

(Example: top margin and right margin in the associated image)

-The rendering of the dotplot updates for a small change of focus (when scrolling when example), which is a bit distracting.

-A panning option, similar to the one available from the linear genome view, would prove useful to navigate the Dotplot viewer.

Linear synteny viewer:

-JBrowse 2 allows exporting views as SVG figures. However, when in the synteny viewer, it seems that tracks from each genome can only be exported independently. An option to export the whole view at once, with tracks from both genomes, would be helpful if the synteny is the focus of the figure.

-I was able to visualize and navigate synteny views for two genomes with no issue, using a PAF file as input. However, I encountered issues when importing .anchors files (from MCScan), even using the same data as provided by the authors. Upon import, the linear synteny view would stay in a continuous "Loading" stage with no other indications. However, zooming out sufficiently (typically, 'Show all regions in assembly') was enough to make it work as expected. The same behavior was observed on Ubuntu and MacOS. This issue is minor but might be worth investigating if it can be replicated by the authors, since it initially led me to believe this import mode was not working correctly..

**Authors Response**

**Point-by-point responses to the reviewers’ comments:**

\## Reviewer 1 response

Thank you very much for the positive review, we greatly appreciate it!

\## Reviewer 2 response

Thank you also for the positive review. This is great feedback, we really appreciate the in-depth look at our project. I will try to respond to the feedback

>The authors benchmarked the performance of JBrowse2 for rendering data using sequencing reads mapped to a reference genome sequence. They note their performances are comparable to IGV.js, another java-script based genome browser. It is arguably true from the data they provide, however, JBrowse2 appears to be more performant at displaying long read data but slightly less performant in rendering short read data than JBrowse 1 and igv.js. This detail does not affect the authors' conclusion but please elaborate on this difference.

In the seven months during which this manuscript has been in review, the performance of JBrowse on short read data has significantly improved. The version in the submitted manuscript was release 1.7.7 while the current release is 2.4.0. The response to this reviewer critique, and more broadly the comparative analysis of JBrowse’s performance to other browsers, is affected by this; accordingly, for this submission we re-ran the entire performance profiling suite and have updated the figures. We have added a note on possible reasons for observed slowness in some cases, for example a slower startup time of the JBrowse 2 app due to having to startup code on the main thread and web worker threads.

>Two references cited from Bioarxiv (JBrowse Jupyter and IGV.js) now have a peer-reviewed paper. Please update it.

Thank you for noting this; we have updated these to the peer-reviewed publications along with another one, Nattestad et al.

>Dotplot synteny viewer: In both the desktop and browser version, this plot sometimes displays long margins at the end of the x axis and y axis when scrolling or zooming in and out, which can result in most of the view being occupied by empty space. (Example: top margin and right margin in the associated image)

Thank you for noting this. Several changes have been made to try to improve this, including smaller margin font sizes, improved zoom behavior using the mouse wheel (which is tangential but improves navigability), and a “Show all regions” option to reset the view.

>The rendering of the dotplot updates for a small change of focus (when scrolling when example), which is a bit distracting.

This is a notable issue, and one we have not yet addressed. We recently fixed a similar issue for our “linear synteny view” (see https://github.com/GMOD/jbrowse-components/pull/3441, no more blinking after scroll) but the analogous change has not yet been added to dotplot. We anticipate that we will be able to address this suggestion in a future version, perhaps as a part of a broader targeted sprint to reduce unnecessary redraws.

>A panning option, similar to the one available from the linear genome view, would prove useful to navigate the Dotplot viewer.

A panning panel, which can be hidden, has been added. We also added mouse-wheel zoom, and click-and-drag to pan the view, which is a more natural google-maps style user interaction.

>Linear synteny viewer: JBrowse 2 allows exporting views as SVG figures. However, when in the synteny viewer, it seems that tracks from each genome can only be exported independently. An option to export the whole view at once, with tracks from both genomes, would be helpful if the synteny is the focus of the figure.

We have now added SVG export to the synteny view (along with all other view types (https://jbrowse.org/jb2/blog/2023/02/24/v2.4.0-release/#svg-export-of-more-view-types)

>I was able to visualize and navigate synteny views for two genomes with no issue, using a PAF file as input. However, I encountered issues when importing .anchors files (from MCScan), even using the same data as provided by the authors. Upon import, the linear synteny view would stay in a continuous "Loading" stage with no other indications. However, zooming out sufficiently (typically, 'Show all regions in assembly') was enough to make it work as expected. The same behavior was observed on Ubuntu and MacOS. This issue is minor but might be worth investigating if it can be replicated by the authors, since it initially led me to believe this import mode was not working correctly.

Thank you for reporting this bug. It has now been reproduced and fixed.
